# A Trigeminoreticular Pathway: Implications in Pain

**DOI:** 10.1371/journal.pone.0024499

**Published:** 2011-09-21

**Authors:** W. Michael Panneton, Qi Gan, Robert S. Livergood

**Affiliations:** Department of Pharmacological and Physiological Science, St. Louis University School of Medicine, St. Louis, Missouri, United States of America; Southern Illinois University School of Medicine, United States of America

## Abstract

Neurons in the caudalmost ventrolateral medulla (cmVLM) respond to noxious stimulation. We previously have shown most efferent projections from this locus project to areas implicated either in the processing or modulation of pain. Here we show the cmVLM of the rat receives projections from superficial laminae of the medullary dorsal horn (MDH) and has neurons activated with capsaicin injections into the temporalis muscle. Injections of either biotinylated dextran amine (BDA) into the MDH or fluorogold (FG)/fluorescent microbeads into the cmVLM showed projections from lamina I and II of the MDH to the cmVLM. Morphometric analysis showed the retrogradely-labeled neurons were small (area 88.7 µm^2^±3.4) and mostly fusiform in shape. Injections (20–50 µl) of 0.5% capsaicin into the temporalis muscle and subsequent immunohistochemistry for c-Fos showed nuclei labeled in the dorsomedial trigeminocervical complex (TCC), the cmVLM, the lateral medulla, and the internal lateral subnucleus of the parabrachial complex (PBil). Additional labeling with c-Fos was seen in the subnucleus interpolaris of the spinal trigeminal nucleus, the rostral ventrolateral medulla, the superior salivatory nucleus, the rostral ventromedial medulla, and the A1, A5, A7 and subcoeruleus catecholamine areas. Injections of FG into the PBil produced robust label in the lateral medulla and cmVLM while injections of BDA into the lateral medulla showed projections to the PBil. Immunohistochemical experiments to antibodies against substance P, the substance P receptor (NK1), calcitonin gene regulating peptide, leucine enkephalin, VRL1 (TPRV2) receptors and neuropeptide Y showed that these peptides/receptors densely stained the cmVLM. We suggest the MDH- cmVLM projection is important for pain from head and neck areas. We offer a potential new pathway for regulating deep pain via the neurons of the TCC, the cmVLM, the lateral medulla, and the PBil and propose these areas compose a trigeminoreticular pathway, possibly the trigeminal homologue of the spinoreticulothalamic pathway.

## Introduction

Pain is an unpleasant sensory and emotional experience associated with actual or potential tissue damage [1]. The *spinothalamic pathway* conveys the conscious perception of pain and has been studied extensively. This pathway carries nociceptive impulses generated peripherally in Aδ and C fibers that project mainly to neurons in laminae I, II, and V of the dorsal horns. These dorsal horn neurons project to the lateral thalamic group for the discrimination of the spatial and temporal dimensions of an injury [2]–[4]. The *spinoreticulothalamic pathway*, also called the paleospinothalamic tract because of its origin in early evolution, mediates arousal as well as the autonomic and emotional aspects of pain. However, this path has been studied little. It is assumed that noxious information conveyed over the spinoreticulothalamic pathway uses similar sensory fibers into the dorsal horn, is relayed to neurons in deeper lamina of the spinal cord, and then ascends to the medulla and pons before projecting to the medial nuclear group of the thalamus [2], [4].

Orofacial pain disorders, comprising 40% of chronic pain afflictions [5], include tension [6], [7] and migraine [8] headaches, cervical myofascial pain syndromes [9], temporomandibular joint disorders [10] and trigeminal neuralgia [11]. Most of these orofacial pain disorders report of increased tension in head and neck musculature and many report changes in autonomic activity [12]. It is thought that many headaches are due to increased tension in head and neck muscles, including the temporalis muscle [13], since electrical stimulation of muscle nociceptors produces an aching pain [2]. Primary afferent fibers innervating the temporalis muscle project centrally both to the dorsomedial medulla via the mesencephalic nucleus and Probst's tract and also to the dorsal horns, especially the dorsomedial parts of the medullary dorsal horn (MDH). [Since the dorsal horns of the medulla and spinal cord share many cytoarchitectural and hodological similarities [14], the dorsal horns of the medulla and the rostral 2–3 segments of the cervical spinal cord collectively are called the *trigeminocervical complex* (TCC) [15].] Those via the mesencephalic nucleus relay information on proprioception while those to the TCC may relay noxious stimuli [16], [17]. A central termination of afferent fibers innervating other muscles of mastication [17], [18] also is in the TCC, and are especially prominent in lamina I and V. Moreover, convergence of input from teeth, muscles of mastication, the TMJ and neck muscles to neurons in the TCC has been noted electrophysiologically [19], [20] and been related to pain. If a function of those convergent neurons in the TCC is related to muscle pain, it may be important in tension headaches.

Neurons in the caudalmost ventrolateral medulla (cmVLM) induce increases in arterial blood pressure when stimulated and the caudal pressor area (CPA) [21]–[23] is included here. Indeed, we [22] noted that relatively small injections of 1 M glutamate into the CPA of anesthetized rats induced abrupt increases in arterial blood pressure but also induced uncontrollable writhing for about 20 sec. Moreover, neurons in the cmVLM also have been implicated in processing of noxious information [24]–[41] and the juxtaposition of pain and cardiovascular pathways noted for this region [23], [29], [32], [42]. In addition, several reports [43]–[46] show neurons “in the white matter ventral to caudalis” [43] responsive to dural stimulation while other studies [47]–[50] noted Fos labeling near the lateral reticular nucleus after noxious stimulation of head structures. The cmVLM thus may indeed be an interface between cardiovascular and pain systems.

Both the anterograde and retrograde tract tracing techniques used herein show a distinct projection from superficial laminae of the TCC to the cmVLM. These techniques also demonstrate the cmVLM, lateral medulla and internal lateral parabrachial nucleus (PBil) are interconnected. The function of this pathway was tested with intramuscular injections of capsaicin, a known algesic agent, into the temporalis muscle and subsequent analysis of neurons using c-Fos immunohistochemistry. We show c-Fos labeling in dorsomedial parts of the TCC, the cmVLM, the lateral medulla, and the PBil. We propose these areas compose a trigeminoreticular pathway regulating deep pain from head and neck regions and may be the trigeminal homologue of the spinoreticulothalamic pathway.

## Materials and Methods

Sprague Dawley rats (275–299 g) were purchased commercially (Harlan Laboratories, Indianapolis, IN, USA) and housed in the Department of Comparative Medicine at Saint Louis University. A protocol (#2063) was approved by the Animal Care Committee of Saint Louis University and followed the guidelines of the National Institutes of Health Guide for Care and Handling of Laboratory Animals. The number of animals used and their pain and suffering were minimized.

Rats were prepared for aseptic surgery after anesthesia with intraperitoneal injections of a mix of ketamine (60 mg/kg) and xylazine (40 mg/kg); additional injections were given as required. They were secured prone in a stereotaxic device (Kopf Instruments, Tujunga, CA, USA) in a flat skull position. Their skull and muscles were exposed via a dorsal incision and their medulla oblongata exposed after cutting the dura. Free micropipettes (20∼25 µm OD) filled either with 10% biotinylated dextran amine (BDA; Molecular Probes, Invitrogen Corp, Carlsbad, CA, USA; 10,000 MW) in saline or 1.5% FluoroGold (FG; Fluorochrome, Inc., Denver, CO, USA) in 0.2 M cacodylate buffer, or micropipettes glued to a 1 µl Hamilton syringe filled with fluorescent microbeads (Lumaflour, Corp., Naples, FL, USA) were lowered into the medulla.

The injections into the caudal medulla were made with the carrier angled anteriorly at 24° from vertical; coordinates for the calamus scriptorius were noted and used as our ‘zero point’. Coordinates for BDA injections into the MDH were varied and depended on the location of the target area. Those for injections of FG or fluorescent beads into the cmVLM were 1.0 mm caudal to calamus scriptorius, 2.0 mm lateral to the midline and 1.7 mm ventral from the dorsal surface of the brainstem. [It should be noted that the calamus scriptorius is situated approximately 650 µm caudal to the rostral edge of the area postrema and 600 µm caudal to the obex, the beginning of the central canal of the spinal cord. Apparently, the calamus scriptorius is labeled the ‘obex’ in a popular rat atlas [51].] Injections of BDA were made into the parvocellular reticular formation of the lateral medulla (LRF) in 5 cases and FG into the parabrachial complex in another 5 cases; these injections used interaural zero as reference.

The BDA and FG tracers were deposited in the brain by passing a positive current (5 mA; 7 s on/off) via a silver wire inserted into the micropipette for 10–15 minutes using a constant current device (MidGuard, Cole-Parmer Instrument Corp, Vernon Hills, IL, USA), while 50–100 nl of fluorescent beads were injected by pressure. The micropipette was left in place for 5 min after all injections. Wounds were washed with sterile saline and closed with silk and wound clips. All rats received subcutaneous injections of the analgesic buprenorphine (0.005 mg/kg) after surgery.

After a survival of 5–10 days, the animals were anesthetized deeply with a Euthanasia solution (IP; Sleepaway, 40 mg/kg) and perfused through the heart using a peristaltic pump first with saline mixed with 0.25% procaine, and then with a fixative of 4% paraformaldehyde in 0.1 M phosphate buffer( PB; pH 7.3). Brains and spinal cords were removed and stored in the fixative with 20% sucrose at 4°C. The brains were blocked in the transverse plane using a precision brain slicer prior to cutting frozen transverse sections (40 µm) with a microtome. A 1∶3 series of sections was collected in PB.

For the BDA cases, sections were washed 3× with 0.1 M PB for 10 min, and then in 0.1 M PB with 0.3% triton for at least 5 min. The sections then were incubated in Vectastain ABC Elite solution (1∶200; Vector Laboratories, Burlingame, CA, USA) for 1 h, washed in 3 rinses of PB, and reacted with diaminobenzidine dihydrochloride (DAB) intensified with nickel ammonium sulfate for 4–10 min. Hydrogen peroxide (0.06%) catalyzed the reaction. Sections containing FG were floated in buffer containing rabbit anti-FG (1∶20,000; Chemicon, Temecula, CA, USA) overnight on a shaker at room temperature. The following morning, the sections were washed in PB with 0.3% triton and incubated for 1 h in a solution containing goat anti-rabbit immunoglobulin (Sigma-Aldrich Corp., St. Louis, MO, USA) at a dilution of 1∶400. After washing in PB containing 0.3% triton, sections were incubated in Vectastain ABC Elite solution (1∶200) for 1 h, and then reacted as above. The sections from all groups were then rinsed, mounted on gelatinized slides and air-dried. Those from BDA and FG cases were counterstained with Neutral Red, dehydrated in alcohols, defatted in xylenes, and coverslipped with Permount.

Rats were anesthetized with ketamine/xylazine as above and a small sterile cannula (PE 50) inserted into their left temporalis muscle 3–5 d before the experiment. The cannulas were secured to the muscle's fascia, tunneled subcutaneously to exit the body on the dorsal neck, and plugged to minimize infection. Initial cases proved that scratching and grooming behaviors were induced after temporalis injections of capsaicin; such behaviors resulted in abundant c-Fos in the brainstem, much of which we considered excessive and spurious. The nine rats reported herein (3 control and 6 experimental) thus were outfitted with an Elizabethan collar (Braintree Scientific Inc., Braintree, MA, USA) for 2 d (2 h/d) and allowed to acclimate to the collars. Such collars prevented the rats from scratching their head and neck regions the day of the experiment. On the day of the experiment, the rats received injections of either normal saline (control group) or 0.5% capsaicin (2–5 µl/min for 10 min) with an infusion pump (Model KDS200; Fisher Scientific, Pittsburgh, PA, USA) into their temporalis muscles via the inserted cannula.

The rats were perfused after 2 h with a 4% paraformaldehyde solution (*vide supra*); their brains and spinal cords removed, and sectioned the next day. Sections were processed immunohistochemically (*vide supra*) with anti-Fos (rabbit polyclonal IgG for c-fos p62; 1∶20,000; Santa Cruz Biotechnology, Santa Cruz, CA, USA), mounted on slides and stained.

Anesthetized rats were perfused, their brains cut frozen, and sections reacted immunohistochemically with antibodies to substance P (rabbit anti-SubP; 1∶5,000; DiaSorin, Inc., Stillwater, MN), leucine enkephalin (rabbit anti-L-ENK; 1∶25,000; Sigma-Aldrich Corp.), substance P receptor (rabbit NK-1; 1∶7,500; Chemicon, Temecula, CA, USA), calcitonin gene-related peptide (guinea pig anti-CGRP; 1∶5,000; Peninsula Laboratories [now Bachem AG; Torrance, CA, USA]), VRL-1 (rabbit anti-VLR-1; 1∶5,000; Chemicon), and neuropeptide Y (rabbit anti-NPY; 1∶12,000; ImmunoStar, Inc., Hudson, WI, USA) as above.

Sections from all experiments were examined with a Nikon E800 microscope equipped with brightfield and fluorescent optics, photographed digitally (MicroImager II, QImaging Corp., Burnaby, BC, Canada), and processed and saved on a computer with Northern Eclipse software (Empix Imaging, Inc., Mississauga, ON, Canada). The labeled fibers in the BDA cases or the location of neurons labeled with FG or c-Fos was reconstructed using a Neurolucida System (MicroBrightField, Inc., Cochester, VT, USA) interfaced with a Nikon E600 microscope. Varicosities on labeled fibers in the BDA cases were considered synaptic boutons, while neurons even minimally labeled with FG or c-Fos were drawn. Neuronal morphology was drawn at a magnification of 200× with the Neurolucida system and data analyzed with NeuroExplorer software. Feret Min and Feret Max were extracted from NeuroExplorer and considered the minimum and maximum diameters of the neurons. Form factor indicates the degree of flatness of the contour; a perfect circle approaches a maximum of 1 while a flatter shape can approach 0. Photomicrographs were adjusted in Adobe Photoshop CS2 software (version 9.0; Adobe Systems, Inc., San Jose, CA, USA) using levels, brightness and contrast, and figures made with Adobe Illustrator CS2 (version 12.0).

The mammalian reticular formation is studied comparatively little and its diffuse nature is problematic for those describing it. Different names are used to describe similar structures/areas while the same name has been used to describe different areas. Since this manuscript describes neurons in several areas of the reticular formation, we elect to define the areas represented by the terminology below.

The term rostral ventrolateral medulla as used herein designates that part of the medulla oblongata up to 800 microns caudal to the facial nucleus bordered dorsally by the compact formation of the nucleus ambiguus. It includes the neurons of the reticular formation ventral to n. ambiguus pars compacta between the medial border of the spinal trigeminal nucleus and lateral border of the inferior olivary nucleus. Thus, included in the rostral ventrolateral medulla is the pressor region (including the C1 adrenergic group of neurons) commonly referred to as the RVLM. The Bötzinger complex of the ventral respiratory column and parts of the rostral semi-compact formation of the ambiguus complex also are here (see Panneton et al., 2005). The unabbreviated term ‘rostral ventrolateral medulla’ will be used when referring to these collective neuroanatomical entities of the medulla.

The term caudal ventrolateral medulla designates the area ventral to the ambiguus complex. The caudal ventrolateral medulla is caudal to the rostral ventrolateral medulla and ends at the spinomedullary junction near the caudal end of the pyramidal decussation and includes the area between the medial border of the spinal trigeminal nucleus and the lateral margin of the inferior olivary nucleus. The caudal ventrolateral medulla near the level of the obex contains the functionally defined cardiovascular depressor area (CVLM), the pre-Bötzinger complex of the ventral respiratory column, and the rostral ventral respiratory group; the caudal ventral respiratory group is caudal to the obex. The A1 group of catecholamine neurons (A1), the nucleus retroambiguus, and the semi-compact, loose and external formations of the ambiguus complex also are found in the caudal ventrolateral medulla. The abbreviation CVLM in this manuscript will designate only the area defined functionally as the cardiovascular depressor area, while the neuroanatomical term “caudal ventrolateral medulla” refers to the larger part of the reticular formation of the ventral caudal medulla.

The term caudalmost ventrolateral medulla (cmVLM) refers to the reticular formation wedged between the lateral reticular nucleus and the MDH near the spinomedullary junction; this area contains both the CPA and the VLMlat, where neurons responsive to noxious stimulation lie [24]. It is unknown if the same neurons are involved in both pressor responses and appreciation of noxious stimuli, or, if functionally-independent neurons are intermixed, but such possibilities have been discussed [42].

The term medullary lateral reticular formation (LRF) as used herein is analogous to that outlined in a rat atlas [51] as the parvicellular reticular nucleus (PCRt) as well as the medullary reticular nucleus dorsalis (MdD). The LRF as defined is analogous to the lateral tegmental fields (LTF) as defined by others [52]. Similar neurons are found rostrally in the caudal pons surrounding the trigeminal motor nucleus, including the nucleus subcoeruleus medially [SubCD and SubCV [51]] and the intertrigeminal nucleus [53] laterally. This latter region is densely innervated after injections in the LRF more caudally (see Results). By these definitions, there is slight overlap of the ventral LRF with the lateral extremes of the rostral and caudal ventrolateral medulla.

## Results

**Figure 1:**
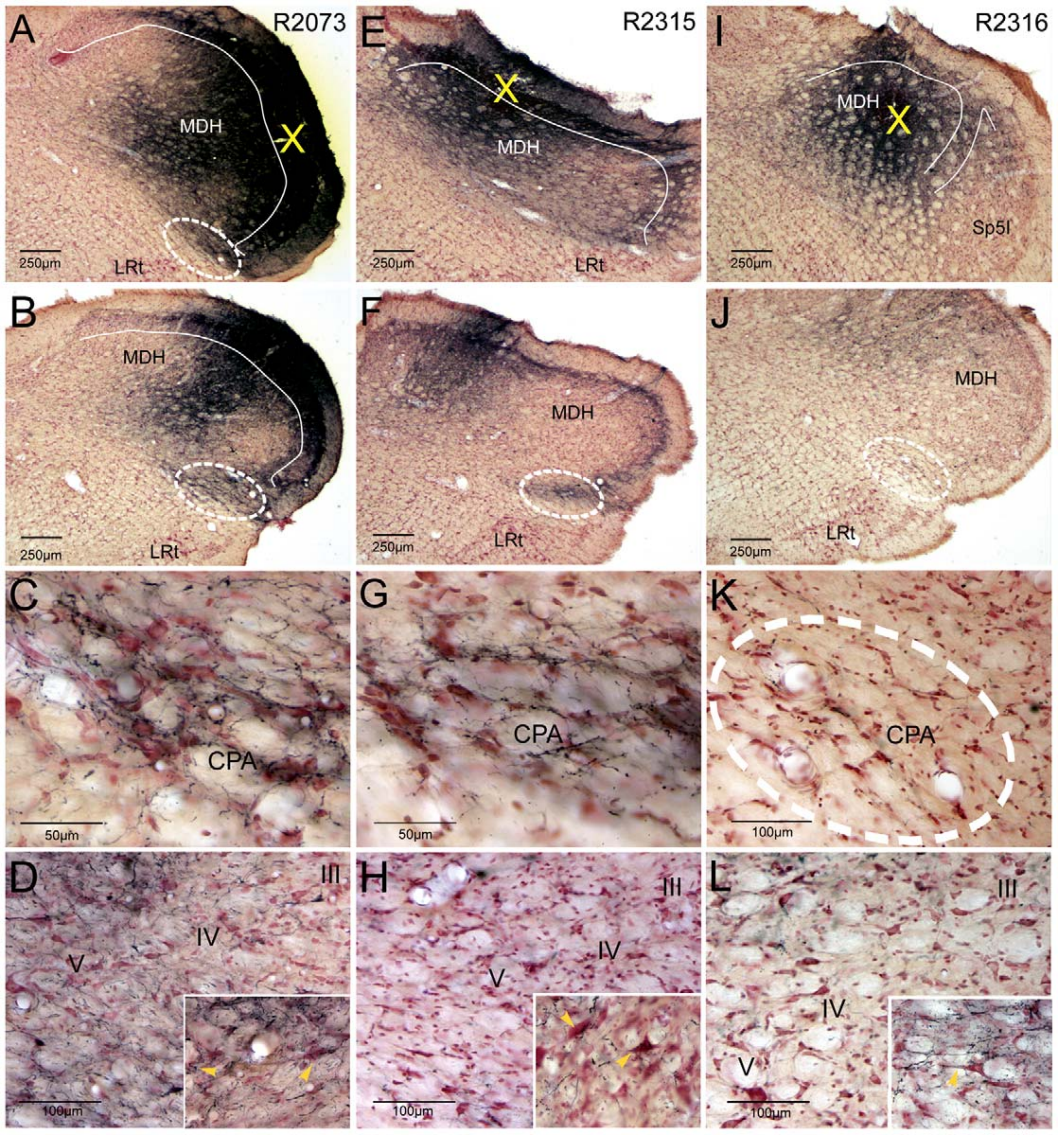
Deciphering projections from the superficial MDH to the caudalmost ventrolateral medulla. Brightfield photomicrographs of brain sections reacted for BDA after its injection into the MDH in three cases. These injections (marked by X in A, E, and I) suggested a projection from neurons in the superficial MDH (laminae I–II) to the cmVLM, including the caudal pressor area (CPA; encircled in A, B, F, J, K). This projection becomes more apparent as the injection center moves from ventrolateral (A) to intermediate (E) to dorsomedial parts of the MDH. Terminal-like label in the cmVLM in B, F, and J is seen in higher magnification in C, G, and K, respectively; note that many small varicosities are associated with these labeled fibers. When injections were centered superficial to the II–III laminar border (white line in A, E), the cmVLM was marked with terminal label (C, G,) but was relatively void of such label (K) with an injection deeper (I). The absence of label in laminae III–IV is seen in D, H, and L, but some large neurons in lamina V were encrusted by boutons (inserts in D, H, L). Encircled area in K marks the CPA. Abbreviation: LRt, lateral reticular nucleus; I, II, III, IV, V, lamina I through V of the dorsal horn. See text for other abbreviations.

Data on anterograde track-tracing with BDA injections in the MDH reported herein were gleaned from over 80 cases. In many of these cases the ventral MDH was targeted, making it difficult to determine a projection to the juxtaposed cmVLM (Fig. 1A; white oval). Other injections directed more dorsally and rostrally (Figs. 1A, E, I; yellow X) however suggested a potential MDH projection to the cmVLM. For example, when injections were placed superficially in intermediate, dorsal or more rostral parts of the MDH (Figs. 1E, I), a gap with minimal labeling of fibers was noted in laminae III–V that contrasted robust label in the cmVLM (Figs. 1B, F; white oval). We also noted that injections centered in laminae III–IV (Fig. 1I) induced no or minimal label in the cmVLM (Fig. 1J). The labeled fibers in the cmVLM always had varicosities, and most were associated with very small fibers (Figs. 1C, G). Fibers with varicosities often were juxtaposed to large neurons in lamina V of the MDH after injections of BDA into superficial laminae (Figs. 1D, H, L; inserts). It also should be noted that retrogradely labeled neurons often were noted in superficial laminae of the MDH after BDA injections centered in the CPA (see insert; Fig. 1D, Sun and Panneton, 2005).

**Figure 2:**
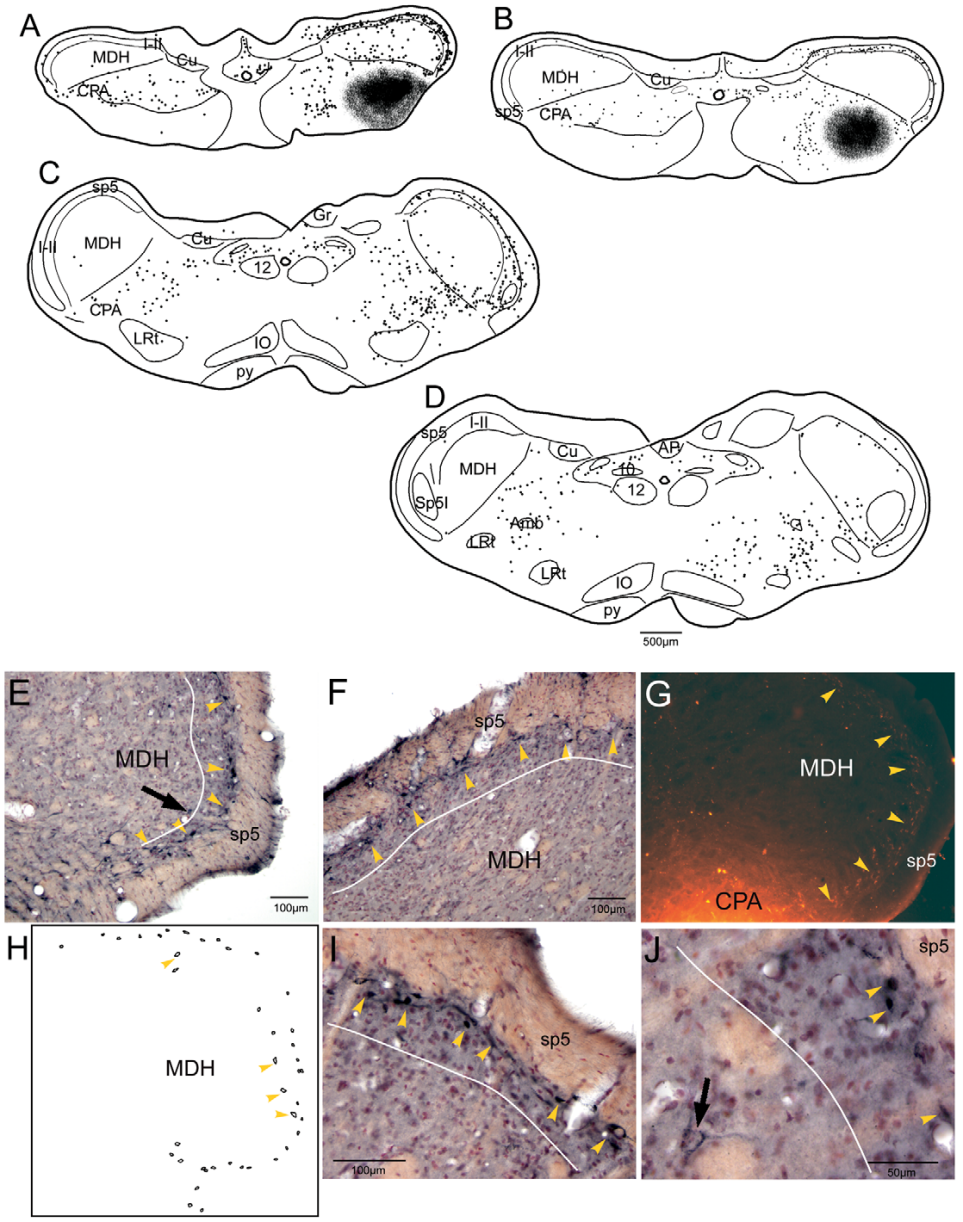
The caudalmost ventrolateral medulla receives projections from neurons in laminae I and II of the TCC. Line drawings and photomicrographs of sections from cases injected with a retrograde tracer in the cmVLM. An injection of fluorogold into the cmVLM (A) resulted in numerous retrogradely-labeled neurons in laminae I and IIo of the ipsilateral MDH (A, B, C), the contralateral cmVLM, including the CPA (A, B, C), and the reticular formation. Numerous retrogradely-labeled neurons in the MDH (E–J) were intensely-stained and small, with dendrites oriented around the curvature of the dorsal horn (yellow arrowheads). Fewer were larger, more lightly-stained, and near the border between lamina II–III (E, J, arrow; H, yellow arrowheads). An injection of fluorescent microspheres, which are not incorporated into fibers of passage, showed similar results (G). Contours of these neurons (H) was used for morphometric analysis. Each dot in A–D represents a single labeled neuron. Abbreviations: Amb, nucleus ambiguus; AP, area postrema; Cu, cuneate nucleus; IO, inferior olivary nucleus; py, pyramidal tract; sp5, spinal tract of the trigeminal nerve; 10, dorsal motor nucleus of the vagus nerve; 12, hypoglossal motor nucleus. See text and previous figure for other abbreviations.

Data on retrograde tract-tracing after FG injections into the cmVLM was taken from 15 cases while 3 cases had injections of fluorescent microspheres. There was no contamination of the MDH after at least three of these FG injections. After such cases, retrograde labeling in laminae I and especially outer parts of II (IIo) was robust in the TCC through the C1 segment (Figs. 2A–D). Most labeled neurons were fusiform, intensely stained, and superficial (Figs. 2E, F, I, J; yellow arrowheads). These neurons seldom displayed long dendrites, but incipient processes usually ran parallel to the curve of the spinal trigeminal tract. Larger, less intensely-labeled neurons (Figs. 2E, J; arrow) were noted near the border of laminae II–III (see Fig. 2H; yellow arrowheads) with dendrites oriented superficially. One hundred twenty three neurons in lamina I–IIo were analyzed for morphology (Fig. 2H). These labeled cells were small, having an area 88.7 µm^2^±3.4, a feret max (maximum diameter) of 15.4±0.37 µm, a feret min (minimum diameter) of 8.2±0.16 µm and a shape factor of 0.76±0.008. Since FG is known to label fibers in passage, however, other cases were done with small injections of fluorescent microspheres into the cmVLM; these microspheres are not known to label fibers in passage. The results of these cases showed numerous neurons labeled in laminae I and II (Fig. 2G, yellow arrowheads), identical to those seen after the FG injections.

**Figure 3:**
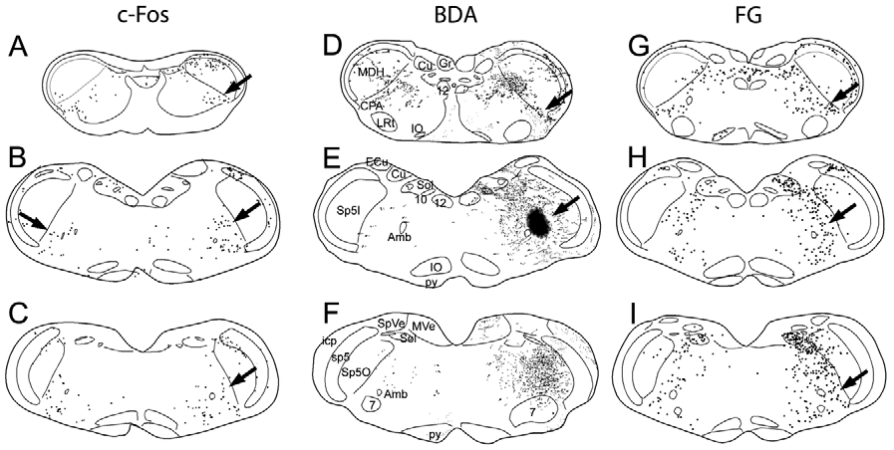
The lateral medulla is implicated in a pathway from the TCC through the caudalmost ventrolateral medulla. Line drawings showing immunoreactivity in the caudal brainstem to c-Fos after a 20 µl injection of 0.05% capsaicin into the temporalis muscle (A, B, C), projections from the injection of BDA into the lateral medulla (D, E, F), and the location of retrogradely-labeled neurons after injections of FG into the PBil (G, H, I). Dorsomedial parts of the MDH were labeled with Fos after temporalis injections of capsaicin (A), as was the CPA in the cmVLM (A, arrow). Since numerous neurons in the LRF were labeled after temporalis injections of capsaicin (B, C; arrows), injections of BDA were made here (E; arrow). Labeled fibers with varicosities spread to the cmVLM caudally (D; arrow) and more rostrally in the LRT (F). Numerous retrogradely-labeled neurons were found in the LRF (H, I) and CPA in the cmVLM (G) after injections of FG into the PBil (see Fig. 5L). Abbreviations: ECu, external cuneate nucleus; Gr, gracile nucleus; Sol, nucleus of the solitary tract; Sp5I, nucleus of the spinal tract of the trigeminal nerve, interpolar part; Sp5O, nucleus of the spinal tract of the trigeminal nerve, oral part; 7, facial motor nucleus; icp, inferior cerebellar peduncle. See text and previous figures for other abbreviations.

**Figure 4:**
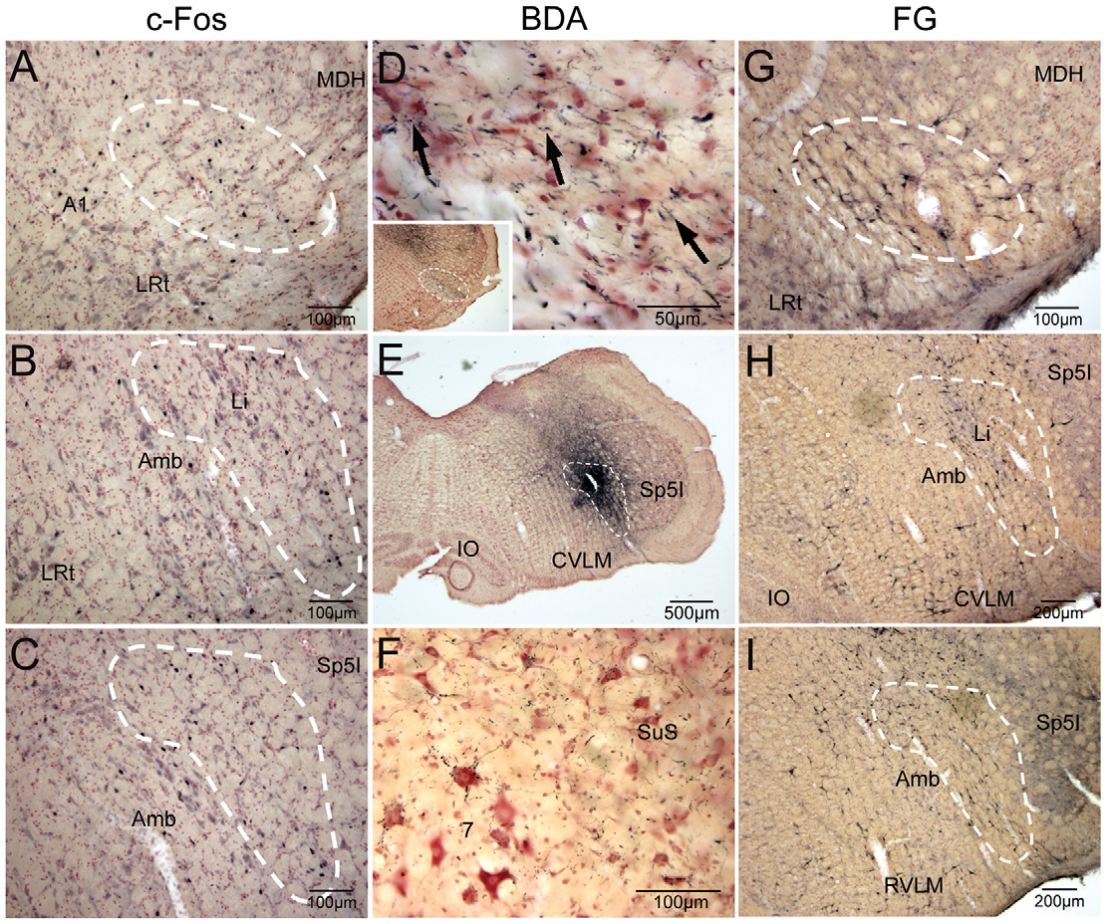
Neurons in the caudalmost ventrolateral medulla and lateral reticular formation are suspected relays of the trigeminoreticulothalamic tract. Photomicrographs of immunolabeling of neurons in the cmVLM to c-Fos after temporalis injections of capsaicin (A; CPA encircled), after BDA injections into the LRF (E; dashed outline) and FG into the PBil injections (G; CPA encircled). The medullary LRF was labeled with Fos immunoreactivity after temporalis injections of capsaicin (B, C; dashed white outline) which mimicked the distribution labeled neurons after the PBil injection of FG (H, I; dashed white outline). The injection of BDA into the LRF also labeled rostral facial motoneurons and the superior salivatory nucleus (F). Abbreviations: Li, linear nucleus of the medulla; SSN, superior salivary nucleus. See text and previous figures for other abbreviations.

**Figure 5:**
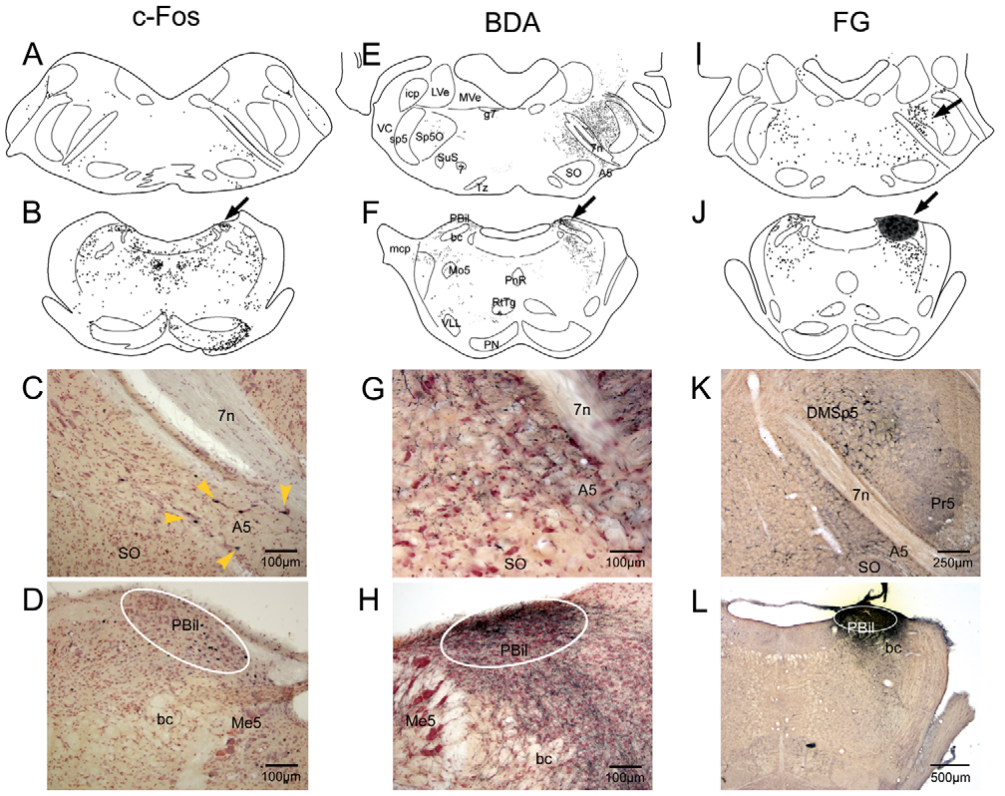
Pontine neurons implicated in pain pathways are labeled with c-Fos, BDA and FG in their respective experiments. Photomicrographs and line drawings through the rostral medulla and pons showing immunoreactivity to c-Fos after capsaicin injection into the temporalis muscle (A–D), fibers and varicosities after an injection of BDA into the LRF (E–F), and retrogradely-labeled neurons after an injection of FG into the PBil (I–L). Note that the PBil is labeled both with c- Fos (B, arrow; D, encircled) and BDA (F, arrow; H, encircled). Labeling with each marker was in the A5 area (A, C, E, G, I, K), while dorsomedial parts of subnucleus oralis were labeled after PBil injections of FG (I, K). Abbreviations: LVe, lateral vestibular nucleus; Me5, mesencephalic trigeminal nucleus; Mo5, motor trigeminal nucleus; MVe, medial vestibular nucleus; PN, pontine nuclei; PnR, pontine raphe nucleus; RtTg, reticulotegmental nucleus; SO, superior olivary nucleus; Tz, trapezoid nucleus; VC, ventral cochlear nucleus; VLL, ventral nucleus of the lateral lemniscus; bc, brachium conjunctivum; g7, genu of facial nerve; mcp, middle cerebellar peduncle; 7n, facial nerve root. See text and previous figures for other abbreviations.

The medullary LRF was targeted with injections of BDA in 5 cases; case R2177 was typical and is described. The injection was relatively small and centered in the LRF just dorsal and lateral to the nucleus ambiguus (Figs. 3E, 4E) with but little spread into the subnucleus interpolaris of the trigeminal sensory complex. Projections were found caudally in the CPA of the cmVLM bilaterally (Figs. 3D, 4D), but little was seen in the MDH except for labeled fibers with swellings juxtaposed to large neurons in lamina V, sporadic retrogradely-labeled neurons in laminae I–IIo, and sporadic fibers with boutons in laminae I–IIo and along the II–III border. There were more numerous labeled fibers and swellings extending from the cmVLM ipsilaterally in a band arching under the nucleus cuneatus, including the subnucleus reticularis dorsalis, and ending in the lateral tip of the nucleus tractus solitarii (Fig. 3D)[called the intermediate reticular nucleus of some [51]]. There was little reaction product in more rostral subnuclei of the trigeminal sensory complex except for dorsomedial parts of the ipsilateral subnucleus interpolaris and subnucleus oralis, which was labeled bilaterally (Figs. 3F, 4E, 5E). The parvocellular LRF throughout the medulla contained numerous small fibers with boutons while the gigantocellular RF more medially also contained similar structures but in lesser amounts (Figs. 3E, F; 4E). Large motoneurons in nucleus ambiguus and the dorsal aspects of the facial nucleus were labeled (Fig. 4F), but little reaction product was seen in the CVLM, the RVLM, or the Bötzinger complex. There were numerous labeled fibers ipsilaterally in dorsomedial parts of the spinal trigeminal tract from unknown origin (Figs. 3F, 5E) as well as the inferior cerebellar peduncle, the latter possibly from inclusion of n. linearis in the injection. In the pons, the A5 area was labeled only moderately (Figs. 5E, G) but the trigeminal motor nucleus and the reticular formation surrounding it contained labeled fibers and boutons (Fig. 5F) as did the PBil (Fig. 5F, H). The ventral and dorsolateral subnuclei of the parabrachial complex, the nuclei locus coeruleus and subcoeruleus, and the A7 area had little or no labeling after this medullary LRF injection, but were sparsely labeled after larger injections. Injections centered medial to n. ambiguus resulted in only sparse label in the cmVLM or PBil, more dorsal injections of the LRF labeled the ventral subnucleus of the parabrachial complex robustly, while larger injections in the LRF, which included medial parts of subnucleus interpolaris, induced much more label in lamina V of the MDH.

Four of the five PB injections of FG were centered in the PBil (Figs. 5J, L) while another was centered in the subnucleus ventralis of the PB. Retrogradely-labeled neurons were found in the cmVLM bilaterally (Figs. 3G, 4G)after PBil injections, as described previously [23]. Moreover, numerous retrogradely-labeled neurons, many of exceptionally large size, were seen in a band extending from the cmVLM through the subnucleus reticularis dorsalis to the lateral part of the nucleus tractus solitarii caudal to the obex (Fig. 3G). More rostrally, the parvocellular LRF contained numerous retrogradely-labeled neurons bilaterally (Figs. 3H, I; 4H, I). We noted that those found more dorsally near the nucleus tractus solitarii appeared smaller than those in the LRF more ventrally. The ventromedial medulla contained retrogradely-labeled neurons, mostly ipsilaterally. The lateral aspect of the gigantocellular RF contained large retrogradely-labeled neurons bilaterally through levels of the facial nucleus but changed to labeled neurons in the dorsal raphe more rostrally. Large neurons in the dorsomedial part of the subnucleus oralis of the trigeminal sensory complex were especially well-labeled (Figs. 5I, K), especially ipsilaterally. The reticular formation surrounding the trigeminal motor nucleus, including the intertrigeminal and subcoeruleus nuclei pars dorsalis and ventralis, were labeled extensively. Reciprocal connections to the PBil on the opposite side are suspected since retrogradely-labeled neurons always were found there (Fig. 5J).

While caudal aspects of the NTS contained medium-large sized neurons labeled retrogradely (Fig. 3G) after PBil injections, smaller neurons were found beginning at the obex and continuing without interruption to the rostral pole of the nucleus (Figs. 3H, I). Many neurons of similar dimensions spilled from the normal nuclear borders of the NTS and occupied the reticular neuropil of the dorsal LRF, blending with the slightly larger neurons found in the LRF more ventrally (Fig. 4I). The ipsilateral trigeminal subnucleus interpolaris had few neurons labeled retrogradely, but the ipsilateral paratrigeminal nucleus was well-labeled retrogradely (Fig. 3H), including the case with injections centered in the PBv, a case which had only occasional ‘casts’ of neuronal labeling in the cmVLM, SRD, and caudal NTS.

**Figure 6:**
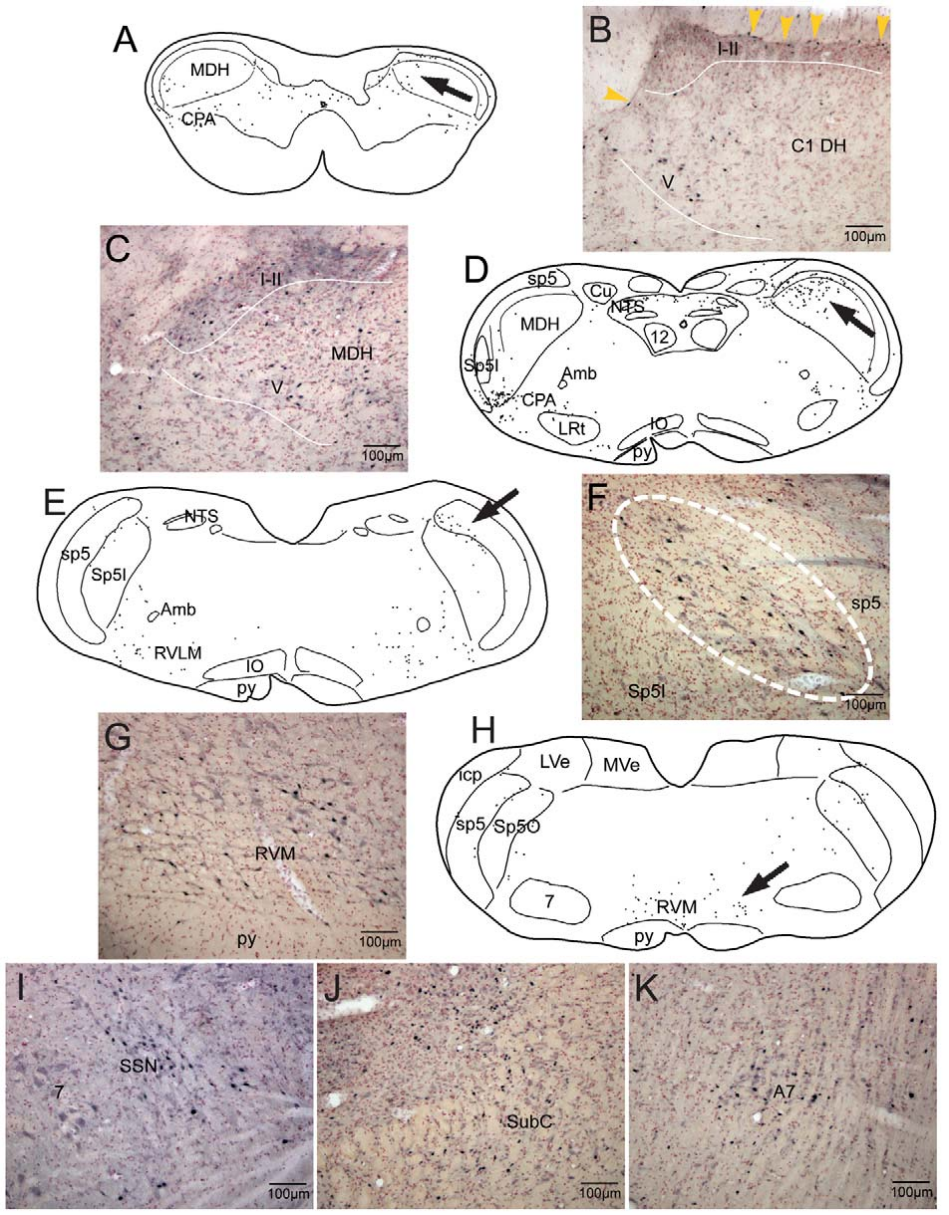
Other areas implicated in pain were labeled after capsaicin injections into the temporalis muscle. Numerous nuclei were immunohistochemically labeled with Fos in lamina I (B; yellow arrowheads) and lamina V of the TCC near the spinomedullary junction (A, B). Similar labeling was seen in the dorsomedial MDH near levels of the calamus scriptorius, but also included neurons in lamina II and III (C; D, arrow). Nuclei were labeled in dorsal subnucleus interpolaris (E, arrow; F, encircled), but their function is unknown. C-Fos profiles were found in the RVM bilaterally (G, H, arrow) dorsal and lateral to the pyramidal tract. The superior salivatory nucleus also was labeled with FOS after temporalis injections bilaterally (I), as were neurons in the subcoeruleus area (J) and A7 area (K). Abbreviations: RVM, rostral ventromedial medulla; SubC, subcoeruleus nucleus. See text and previous figures for other abbreviations.

An injection of 0.5% capsaicin was infused over 10 minutes into the temporalis muscle to induce pain. After such injections, numerous profiles immunoreactive to c-Fos were found in laminae I and V of dorsomedial parts of the TCC (Figs. 3A; 6A–D) ipsilateral to the injection. Immunoreactive nuclei continued rostrally into dorsomedial parts of the ipsilateral MDH and now included laminae II and III near levels of the calamus scriptorius (Fig. 6D). Moreover, numerous nuclei labeled immunohistochemically with c-Fos antibodies were seen in the cmVLM coincident with the CPA (Figs. 3A, 4A, 6D). These labeled nuclei were found bilaterally but with an ipsilateral preponderance. Immediately caudal to the obex, immunolabeled nuclei were found bilaterally at the ventromedial tip of the trigeminal complex, an area labeled the Vi-Vc transition zone (Fig. 6D). Labeled neurons extended from this area into lamina I in the ventral MDH. Above the obex, most immunoreactive profiles were in the LRF. Most such label was caudal to the compact formation of nucleus ambiguus (Figs. 3B, C, arrows; 4B, C, white outlines), but some continued bilaterally to the level of the facial motor nucleus. Numerous profiles also were noted in the rostral ventrolateral medulla (Figs. 3C, 6E), including the RVLM.

An abundance of darkly-stained large nuclei were seen in all cases bilaterally in dorsal parts of the rostral subnucleus interpolaris within or juxtaposed to the spinal trigeminal tract, but mostly on the side of the capsaicin injection (Figs. 6E, arrow; 6F, white oval). The rostral ventromedial medulla (RVM) always was labeled bilaterally after temporalis muscle injections, from the rostral pole of the inferior olivary complex to levels of the trapezoid nuclei (Figs. 6G; 6H, arrow). [The term RVM as used herein includes the pars alpha, gigantocellular ventralis and lateral paragigantocellular nuclei.] Nuclei immunoreactive to c-Fos were found bilaterally in the superior salivatory nucleus (Fig. 6I), and larger labeled nuclei were seen in the A5 area (Figs. 5A, C). Labeling was noted in the external lateral and dorsal lateral subnuclei of the parabrachial complex of the dorsolateral pons (Fig. 5B) in some cases, while considerably more immunoreactive profiles were seen in its PBil in all cases (Figs. 5B, D).

Areas known to contain catecholamine neurons (especially those in the A1, A5, subcoeruleus, and A7 groups; Figs. 6J, K) had numerous immunoreactive profiles. While c-Fos distribution mimicked those of catecholamine neurons in these areas, no double labeling procedure was done for the present study. There were some immunoreactive profiles also in the nucleus of the solitary tract, a few cells around the ventral periphery of the nucleus cuneatus, e.g. the subnucleus reticularis dorsalis. There also were sporadic immunoreactive neurons in ventral parts of lamina III-IV of the MDH bilaterally, the caudal ventrolateral reticular formation, ventral cochlear nucleus, the vestibular nuclei, the reticulotegmental nucleus and pontine central grey matter.

**Figure 7:**
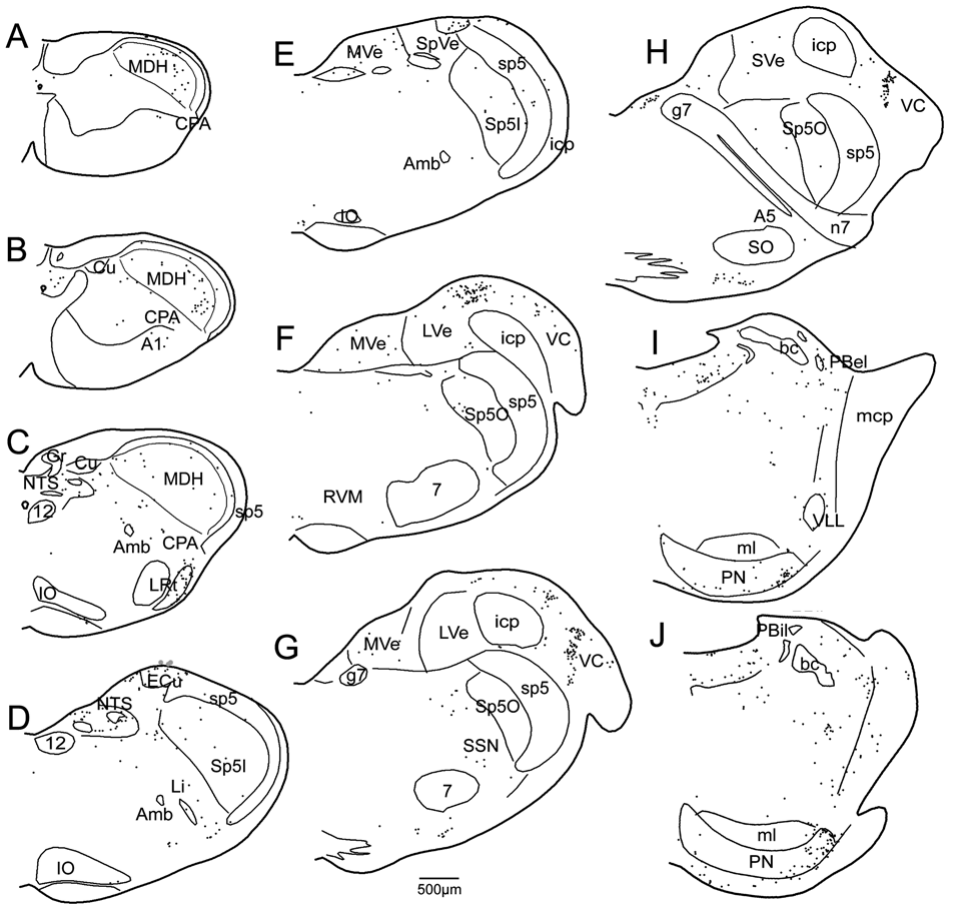
Sparse c-Fos labeling was seen in control rats. Line drawings of brainstem sections showing nuclei immunoreactive to c-Fos after an injection of saline into the temporalis muscle of a rat. Note the minimal c-Fos label in dorsomedial parts of the MDH (A, B, C), the CPA in the cmVLM (A, B, C), lateral medulla (D, E), RVM (F), A5 area (H), and the external lateral, dorsal lateral and internal lateral subnuclei of the parabrachial complex (I, J) from this control animal. Compare to figures 3 and 4. Abbreviations: SpVe, spinal vestibular nucleus; SuVe, superior vestibular nucleus; ml, medial lemniscus. See text and previous figures for other abbreviations.

Labeling in control cases was relatively sparse (Fig. 7). Immunoreactive profiles sometimes were seen bilaterally in laminae III–IV of the TCC, but these generally were found ventrally. We considered these profiles the result of the rat's fur rubbing against the Elizabethan collar. Label in other areas, including the vestibular nuclei, ventral cochlear nuclei, lateral reticular nucleus, pontine nuclei, and reticulotegmental nucleus, and pontine grey appeared to be random and has been noted in other studies [54], [55]. It should be noted however that immunoreactive labeling for c-Fos in dorsomedial parts of the MDH, cmVLM, LRF, RVM, A5 area, and the PBil was unique to cases in which capsaicin had been injected into the temporalis muscle.

**Figure 8:**
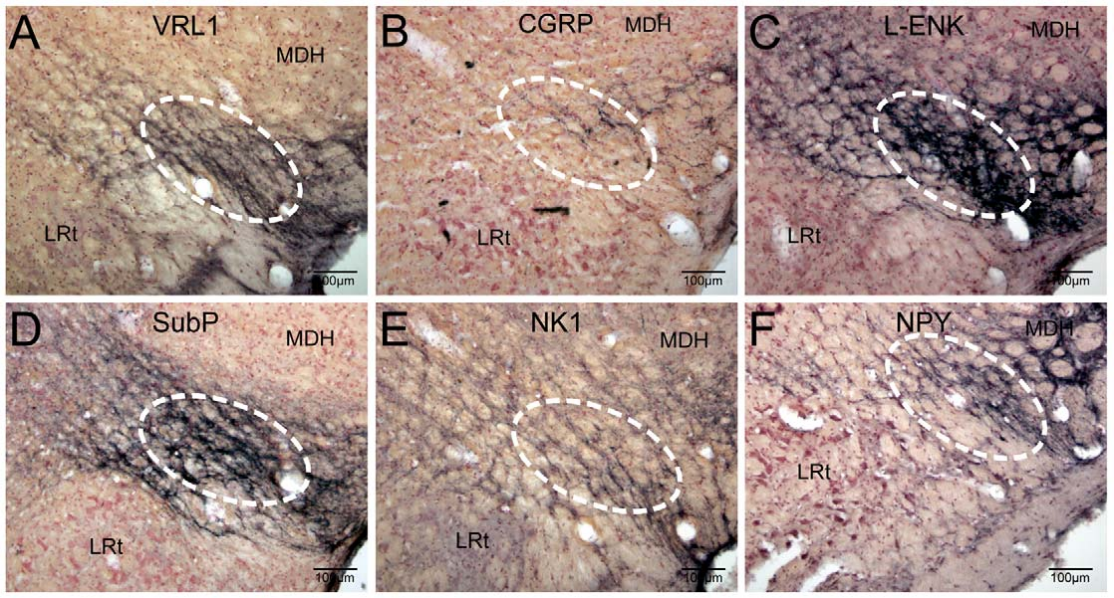
The caudalmost ventrolateral medulla exhibits numerous peptides associated with pain pathways. Photomicrographs through the cmVLM showing immunoreactivity to multiple peptides (white oval encircles the CPA). All these peptides have been implicated in the processing of painful information. See text for discussion and abbreviations.

Immunohistochemical studies revealed dense staining in the caudalmost ventrolateral medulla against antibodies for VRL1 (Fig. 8A), CGRP (Fig. 8B), L-ENK (Fig. 8C), SubP (Fig. 8D), the NK1 receptor (Fig. 8E), and NPY (Fig. 8F). Numerous studies have shown these peptides to be important in the transmission of nociceptive information.

## Discussion

**Figure 9:**
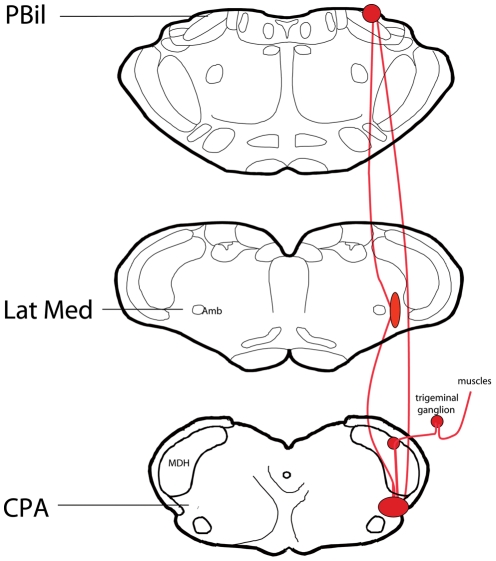
A trigeminoreticular pathway as a route for ascending noxious information. Summary diagram illustrating the presumptive pain pathway from the TCC to the cmVLM and continuing through the LRF and PBil. We propose this is the initial part of a paleo-reticulo-thalamic pathway of the trigeminal system and suggest it may be important for transmitting deep pain from head and neck regions.

Neuroanatomical experiments demonstrated a robust projection from neurons in laminae I and II of the TCC into the cmVLM. Similar experiments also documented projections between the parvocellular LRF and the parabrachial complex, particularly its internal lateral subnucleus. Injections of capsaicin into the temporalis muscle of rats induced c-Fos labeling in the dorsomedial part of the TCC, the cmVLM, the LRF, and the PBil. We propose these areas are directly connected and form a pathway from the TCC through the cmVLM-LRF-PBil (Fig. 9) to the medial thalamus, and are important for mediating deep pain from head and neck regions. This pathway may be the trigeminal homologue of the spinoreticulothalamic pathway, considered to be important for the autonomic and emotional aspects of pain.

There is limited distance between the MDH and the cmVLM and inclusion of both structures in any injection is problematic. However, the TCC is relatively large and allows injections confined to only its superficial laminae. Injections into more dorsal parts of the superficial MDH never included the cmVLM and were separated from it by unlabeled deeper lamina of the MDH (see Fig. 1). Injections of BDA however label both projection neurons as well as neurons with local connections, impeding one's determination of injection extent. This is especially true for injections in the MDH, where intrinsic neurons have numerous interactions with their neighbors. Both BDA and FG can be incorporated into intact fibers of passage [56], [57] and could have induced spurious labeling. Fortunately the fluorescent microspheres used herein neither spread nor label intact fibers of passage, and injection size can be controlled by the volume injected [56]. Moreover, the fluorescent beads produced identical results to the FG injections. Since the small neurons in lamina II have a nuclear∶cytoplasmic ratio approaching unity, many neurons retrogradely labeled in the TCC after injections into the cmVLM were marked by few intracellular inclusions. Both anterograde and retrograde techniques also were used to document projections from the medullary LRF to the PBil. Fluorescent beads were not used in this phase since the distance between these targets is greater and the PBil can be considered in peripheral parts of the reticular formation, thus not subject to the innumerable fibers of passage problematic more ventrally.

Capsaicin is considered to activate only small diameter C-fibers, and such activation is dose dependent [58], [59]. Most of the injections used herein were only 20 µl. Numerous psychophysical studies [60]–[65] have shown that intramuscular injections of capsaicin induce a dull pain in humans and commonly stimulate fibers of small diameter in muscles of experimental animals. Studies in humans have noted that pain fades 20–30 min after a single bolus injection of capsaicin [66], [67] and the deep muscle pain often is referred [64], [67]. c-Fos is an immediate-early gene thought to alter long-term cellular function [68]. Immunohistological detection of Fos, the protein product of the c-Fos gene, often is used as a marker of neuronal activation [69] and effectively measures parts of a reflex circuit if done properly [70]. This technique is limited, however, since production of Fos protein indicates that a neuron has been activated, but not the function of that neuron. Thus any neuron that is part of a reflex circuit could have an afferent, efferent, or integrative function. Additionally, Fos may not be expressed equally in all neurons [70] and neurons that are inhibited do not express Fos.

Although the oral end of the organism offers unique terminal fields for study of somatosensation, investigations of head and neck pain are less frequent compared to pain mediated through the spinal cord. Nevertheless, all head and neck pain is thought to be first processed in the TCC. While it is well-known that projections mediating pain from neurons in laminae I and V of the dorsal horns are important in the spinothalamic pathway, few neurons in lamina II are known to project outside the dorsal horn and most are considered local circuit neurons by many [71]–[73].

Our BDA experiments indicate a dense projection from neurons in superficial lamina of the TCC to the cmVLM. Neurons in laminae I and IIo are well-documented as receiving small diameter primary afferent fibers responsive to noxious stimulation. Their projection to the cmVLM provides another route by which noxious stimuli may exit the dorsal horn for ascension to areas important for either the appreciation or modulation of pain. This projection to the cmVLM may be analogous to those previously described from lamina I neurons in the spinal cord responsive to noxious stimulation to the cmVLM [35], [74], [75]. Numerous TCC neurons in laminae I and II were labeled retrogradely herein after injection of a retrograde tracer into the cmVLM. Many of the retrogradely-labeled neurons were similar to the central neurons of Cajal of the substantia gelatinosa [72] or fusiform neurons described in lamina I of the spinal dorsal horn [3], [35], [76]–[80], some of which project to the cmVLM [3], [74], [81]. Our injections must have been considerably smaller than those in the Lima et al. study (1991) however, since we saw very little labeling in laminae III and IV.

The proposed trigeminoreticular pathway for the transmission of chronic and deep pains needs by definition components in the reticular formation. We propose neurons in both the PBil and LRF as potential candidates for this role. Numerous neurons in the parabrachial complex are responsive to noxious stimulation. Subnuclei in lateral parts of the PB complex, many of which project to the extended amygdala, receive projections from numerous neurons in lamina I of the medullary and spinal dorsal horns [75], [82]–[87] and are responsive to nociceptive stimuli [88]–[93]. More medially and rostrally, the PBil receives dense projections from the CPA in the cmVLM [23] and deep laminae of the spinal cord [84], [87], [94]. Most neurons in the PBil are responsive to noxious stimulation of large body fields [95] and project to the intralaminar thalamus [95], [96]. Herein we also show that the LRF has numerous projections to the parabrachial complex, especially PBil, using both anterograde and retrograde methodologies.

The c-Fos technique allows for visualization of activated neurons along a multisynaptic pathway; it is especially relevant that proper stimulation be done to reduce distractions produced by activating non-relevant systems. Visualization of activated neurons to a noxious trigeminal stimulus has been done using the c-Fos technique [97]–[101], including the masseter muscle and temporomandibular joint [37], [39], [102] and the cornea [102]–[106]. While some c-Fos immunolabeling is reduced by blocking transmitters [105], [107]–[109] or either enhanced [103] or reduced [37] by adrenalectomy, all these reports showed somatotopically-appropriate labeling in laminae I–II of the TCC as well as labeling in ventromedial parts of the trigeminal sensory complex near the subnucleus interpolaris/MDH (the Vi-Vcjunction). The results of the present study also show Fos immunolabeling in somatotopically-appropriate areas of the MDH as well as the ventromedial Vi-Vc transition zone after capsaicin injections into the temporalis muscle.

The Vi-Vc transition zone is especially problematic since noxious stimulation of numerous somatotopically-different receptive fields of the head activate these neurons, leading some authors to suggest they play an important role for transmitting deep pain rostrally [110]–[112]. The ventral Vi-Vc transition zone apparently receives primary afferent projections from the ipsilateral masseter muscle, since trigeminal ganglion cells are double-labeled after injections of retrograde tracers here [112]. A similar area is densely innervated by primary afferent fibers innervating the anterior ethmoidal nerve (AEN) [83], which innervates anterior parts of the nasal mucosa and vestibule, as well as the cornea [see [113] for references], both of which are innervated by an abundance of small diameter fibers [114]. We have shown the central projections of the AEN, however, continue dorsally and laterally from this transition zone and follow the rostral aspect of the substantia gelatinosa as it transposes into the subnucleus interpolaris. This line is similarly marked by numerous peptides which innervate superficial laminae of the MDH; it probably contains neurons similar to those found in both laminae I and II in the laminated part of the MDH more caudally. Nuclei immunolabeled with Fos also follow this same line during forced underwater submersion of rats [54], but not voluntary diving, adrenalectomized rats with their masseter muscles injected with complete Freud's adjuvant [37] and noxious pinch of the nose and interramal vibrisse [97]. Some of these neurons project to more rostral nuclei implicated in pain pathways, including the ventroposteromedial nucleus of the thalamus, the parabrachial complex, the lateral hypothalamus, the rostral ventromedial medulla and especially the nucleus submedius of the thalamus [110], [111].

However, animals from all but one of the studies mentioned above were anesthetized with various anesthetics prior to stimulation and some had cutaneous incisions/removals before stimulating deep structures; both these procedures are known to induce spurious labeling. Indeed, the ventromedial Vi-Vc transition zone is shown labeled in anesthetized animals without stimulation [37], [97], [110], suggesting labeling here may be due to anesthetic. However, the present rats were awake and received no anesthetic, yet the ventromedial Vi-Vc transition zone was labeled bilaterally, suggesting such labeling was not anesthetically-induced. While the function of these neurons remains an enigma, most data suggests they are activated by noxious stimuli but perhaps perform functions other than the perception or modulation of pain. The brainstem and spinal cord also are the loci of numerous reflex circuits and perhaps Vi-Vc neurons are interneurons of one such pathway [115].

Clenching of the teeth requires contraction of the temporalis muscles and is often a source of tension headaches. The c-Fos immunolabeling seen in the dorsomedial MDH approximates the termination of fibers innervating the temporalis muscle in the cat [17], suggesting the injection of capsaicin activated C-fibers innervating this muscle. Many neurons immunolabeled with c-Fos also were found in the cmVLM after injections of capsaicin into the temporalis muscle, supporting other data (*vide supra*) that neurons in this area are responsive to noxious stimuli.

Less attention has focused on the LRF in regards to nociceptive stimuli, but we found numerous neurons labeled with c-Fos here after temporalis injections of capsaicin as well as with FG after PBil injections. Other studies using c-Fos also described immunoreactive neurons in the lateral medulla below the obex after trigeminal stimulation activating nociceptors, and two show them more rostrally at the level of the CVLM [37]. The nuclei labeled herein with c-Fos in the dorsal subnucleus interpolaris were large and bilateral. The function of these neurons is unknown but similar neurons in this area receive both direct projections from the MDH after injections of BDA and are labeled transganglionically after applications of herpes virus to the AEN [116]. c-Fos labeling was found herein in both the rostral ventrolateral medulla and the superior salivatory nucleus and activation of these neurons may modulate autonomic responses to a painful stimulus. It still must be determined if the LRF neurons projecting to the PBil are responsive to nociceptive stimuli, however.

Several areas known important for the descending control of pain also were activated after temporalis injections of capsaicin. The RVM showed abundant c-Fos label and has long been known to modulate pain systems. Neurons immunolabeled with c-Fos were noted in areas where the A1, A5 and A7 catecholamine groups are located and the A5 and A7 areas are considered part of a descending pathway modulating nociception. Future double-labeling experiments will test whether these neurons labeled with c-Fos are indeed catecholaminergic. We propose that activation of neurons in the cmVLM, LRF, and PBil identifies an ascending pain pathway relaying to the medial thalamus and cortex which is important for the appreciation of deep pain emanating in the head.

Although we give little credence to assigning a function to a neural transmitter, several peptides commonly are associated with nociceptive pathways. SubP and CGRP are well-known transmitters in small primary afferent fibers, many of which carry information about noxious stimuli into superficial laminae of the dorsal horn. L-ENK and SubP are found throughout the CNS and have been implicated in numerous studies associated with pathways important in pain. While CGRP, Sub P and L-ENK are involved in pain pathways, neuropeptide Y has received lesser attention but may be important in neuropathic pain in tension-like headache [117]. Previous data has shown that the CPA receives direct primary afferent projections from the anterior ethmoidal nerve [83], and that projections of sensory fibers immunoreactive to CGRP are lost in the CPA after trigeminal rhizotomy but SubP immunolabeling is maintained [118].

Neurons immunoreactive to VRL1, L-ENK, and the SubP receptor (NK1) are in lamina II [119]–[128], and fibers with varicosities of similar substances are presented herein in the cmVLM. The VRL1 antibody is against a protein homologous to TPRV2 and encodes for an ion channel protein in the membranes of Aγ and C fibers [129]. VRL1 is activated by high temperature stimuli [130] while VR1 is a receptor activated by capsaicin, acidic solutions, or moderate heat, and is found on polymodal nociceptors [131], [132]. Neurons in lamina II also are immunoreactive to VR1 [133]. Nevertheless, the source of the peptides reported herein in the cmVLM is unknown, but some could emanate from the TCC in the pathway we have described.

We show herein projections from superficial laminae of the TCC to the cmVLM, from the LRF to the PBil, and activation of neurons in the MDH, the cmVLM, LRF and PBil after injection of capsaicin into the temporalis muscle (see Fig. 9). The fact that stimulation of a similar area (the CPA) induces an increase in arterial blood pressure emphasizes the relation of blood pressure and pain sensitivity [134]. We [23] have shown major projections from the CPA to both the subnucleus reticularis dorsalis (SRD) and the caudal nucleus tractus solitarii (NTS), both of which are important in pain modulation. Moreover, neurophysiological data on the SRD note these neurons respond to noxious stimulation of large receptive fields [135]–[139], similar to neurons in the cmVLM.

It has been speculated that the spinoreticulothalamic pathway mediating pain relays in neurons in the lateral reticular nucleus, gigantocellular reticular formation, and nuclei pontis oralis and caudalis [2] but we have not found any studies that have shown this experimentally. The present study found no neurons labeled with c-Fos in these areas of the medullary reticular formation after capsaicin injections into the temporalis muscle. However we previously have shown relatively dense projections via very small fibers to the LRF from the CPA [23]. The lateral medulla is known to modulate sympathetic activity [140]–[142], and has been implicated in pain [143], [144]. Moreover, clinical literature suggests the lateral medulla may be important in chronic ‘reticulothalamic’ pain in the Wallenberg syndrome [143], [144]. We propose neurons of the LRF may be a reticular relay for a pain pathway, including those important for headache, via a projection from the cmVLM.

A major projection of the CPA is to the PBil [23]. Neurons in the PBil also have been characterized neurophysiologically as responding only to noxious stimulation of very large receptive fields [91], [95], similar to neurons in the cmVLM. The projections of the PBil are to the intralaminar nuclei of the thalamus [96], which then projects to cortical areas, including the prefrontal and insular cortex. Both areas are activated in chronic pain syndromes in humans and have been implicated in the ‘emotional’ aspects of pain (see Bourgeais et al., 2001). Nonetheless, victims of deep pain generally describe their pains as diffuse, aching or throbbing versus the well-localized sharpness of fast pain. The diffuse nature of deep pain may imply a convergence of projections from the TCC to the cmVLM and implicate this area as a relay important for deep pains in the head and neck. Future experiments will explore the significance of neurons in the lateral medulla and parabrachial complex for their roles in the pain experience.
